# Complicated Biliary Stenosis Following Blunt Abdominal Trauma: A Case Report

**DOI:** 10.7759/cureus.109708

**Published:** 2026-05-26

**Authors:** Amanda Tollini de Moraes, Bruno Cordeiro Napoli, Marcos Antonio Marton Filho

**Affiliations:** 1 Department of Medicine, Faculty of Medicine, University of São Paulo, São Paulo, BRA; 2 Department of Surgery, Bauru State Hospital, Bauru, BRA; 3 Department of Medicine, Bauru Medical School, University of São Paulo, Bauru, BRA

**Keywords:** acute pancreatitis, bile duct stenosis, biloma, case report, ercp, hemorrhagic shock, pleural effusion, trauma

## Abstract

Bile duct injuries following blunt abdominal trauma are rare complications that may go unnoticed during emergency care. Due to edema and inflammation, these injuries can lead to bile duct obstruction, resulting in dilation of the hepatobiliary system and potential perforations. Such cases are medical emergencies that require prompt and effective management. This case report aims to present a rare case of bile duct stenosis secondary to blunt abdominal trauma, managed using a minimally invasive approach. Outcomes following endoscopic retrograde cholangiopancreatography (ERCP) are also described. A 25-year-old male patient was admitted with progressive right upper quadrant abdominal pain, accompanied by nausea, vomiting, jaundice, choluria, and acholic stools, occurring two months after sustaining blunt abdominal trauma in a car accident. The trauma had resulted in liver and pancreatic injuries, which were managed conservatively. Imaging revealed bile duct dilation and an abrupt transition in the caliber of its distal third, suggestive of biliary injury. Due to obstructive jaundice, the patient underwent ERCP, which confirmed significant bile duct stenosis. A biliary sphincterotomy was performed, and a prophylactic pancreatic stent was placed to reduce the risk of post-ERCP pancreatitis. However, the patient subsequently developed acute pancreatitis and hemorrhagic shock. Upper gastrointestinal endoscopy identified bleeding and sclerosis of the duodenal papilla. Endoscopic hemostasis using clips, bile duct drainage, and placement of a biliary prosthesis were performed, resulting in clinical improvement. Seventeen days later, the patient developed hospital-acquired pneumonia with pleural effusion and underwent thoracostomy with closed pleural drainage, along with antibiotic therapy using piperacillin and tazobactam, which resolved the condition. The patient remains under outpatient follow-up, undergoing serial dilation of the bile duct. Bile duct strictures are rare complications of blunt abdominal trauma and can have serious consequences. Therefore, it is essential for healthcare teams to remain vigilant for potential biliary tract involvement in post-traumatic patients, maintaining a high index of suspicion in the presence of clinical deterioration or symptoms of biliary obstruction.

## Introduction

The liver is among the most frequently injured solid organs in blunt abdominal trauma. While penetrating trauma, such as stab and gunshot wounds, accounts for approximately 80% to 90% of biliary injuries, biliary tract injuries caused by blunt trauma, primarily resulting from motor vehicle accidents, kicks, and falls, are rare, occurring in only 2.8% to 7.4% of cases [1].

Liver injuries caused by blunt trauma may go unnoticed during emergency care due to associated intra-abdominal injuries. Edema and inflammation from these injuries can lead to obstruction and stenosis of the bile ducts, resulting in hepatobiliary duct dilation and potential perforations, which are medical emergencies requiring prompt and effective management [2].

The clinical manifestations of these injuries vary depending on the underlying causes, but typically include jaundice, choluria, and fecal acholia due to the obstructive process. In such cases, endoscopic retrograde cholangiopancreatography (ERCP) and the placement of a pancreatic prosthesis are among the primary recommendations. However, even with successful management, severe postoperative complications may occur [1,2].

This report describes the clinical course of a patient who developed bile duct stenosis following blunt abdominal trauma in a car accident, along with complications arising from its management.

## Case presentation

A 25-year-old male patient was involved in a high-energy motor vehicle accident. During the initial trauma assessment in the emergency setting, liver and pancreatic injuries were identified on imaging and managed nonoperatively due to the patient’s hemodynamic stability. Approximately two months after the traumatic event, the patient presented to our service through the emergency department with progressive right upper quadrant abdominal pain, accompanied by nausea, vomiting, jaundice, choluria, and acholic stools.

Upon admission, the patient underwent laboratory evaluation, which revealed elevated total and direct bilirubin levels, consistent with obstructive jaundice. He also underwent magnetic resonance imaging (MRI) (Figure 1) and magnetic resonance cholangiopancreatography (MRCP) (Figure 2). Imaging demonstrated a cystic hepatic lesion measuring 6.6 cm, containing internal fluid with debris. Although imaging findings were suggestive of a biloma, the differential diagnosis included seroma and hematoma, and, less likely, pancreatic pseudocyst. The diagnosis of biloma was supported by its anatomical relationship with the biliary tree and was later confirmed following percutaneous drainage. Additionally, dilation of the common bile duct, measuring up to 1.3 cm, was observed, with an abrupt caliber transition in its distal third, suggestive of biliary obstruction. Diffuse dilation of the intrahepatic bile ducts, measuring up to 0.8 cm, was also noted.

**Figure 1:**
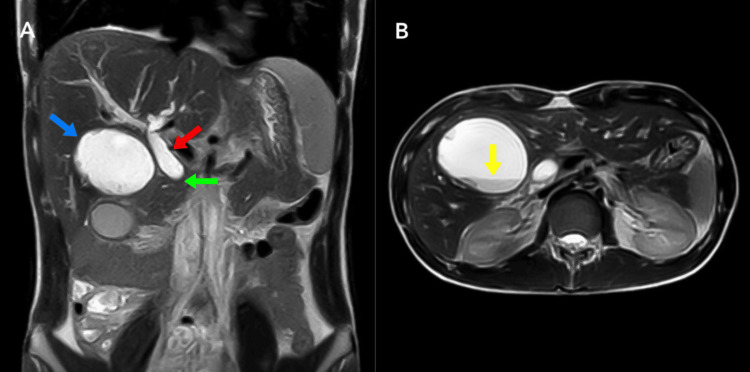
T2-weighted magnetic resonance imaging of the abdomen. A: Coronal section showing a hepatic cyst (blue arrow) adjacent to the dilated hepatocholedochus, with a caliber of up to 1.3 cm (red arrow), and an abrupt narrowing in its distal third, indicative of biliary stricture (green arrow). B: Axial section showing a hepatic cyst measuring 6.6 cm at its largest axis, containing internal fluid deposits (yellow arrow).

**Figure 2:**
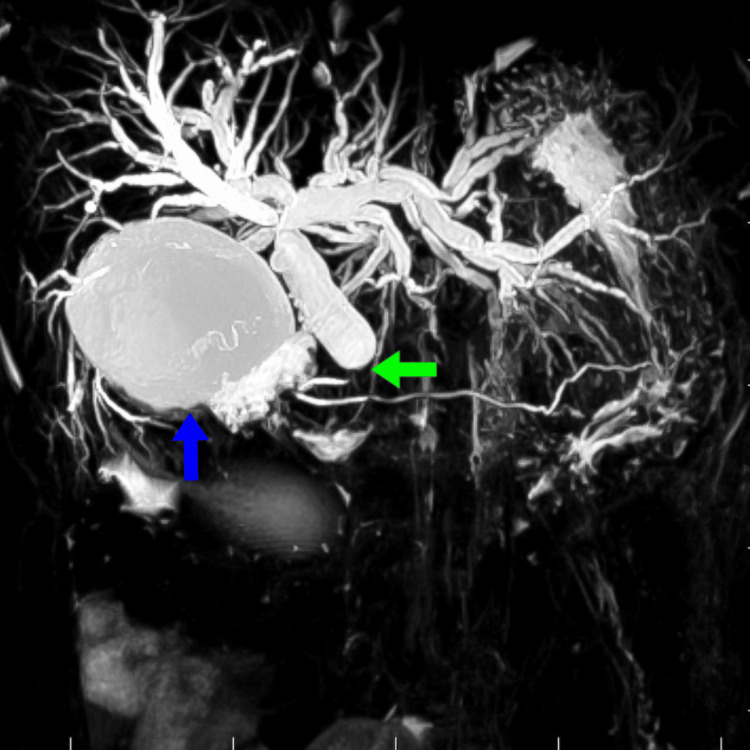
Magnetic resonance cholangiopancreatography performed using the HASTE (half-Fourier acquisition single-shot turbo spin-echo) sequence, with three-dimensional reconstruction in multiple planes. Hepatic cyst (blue arrow) containing internal deposits, measuring 6.6 cm, centered on segment V, suggestive of a bile duct origin. Dilation of the hepatocholedochal canal, showing an abrupt narrowing in its distal third, indicative of biliary stenosis (green arrow). Diffuse dilation of the intrahepatic bile ducts, with a caliber of up to 0.8 cm. Hydropic gallbladder with thin, smooth walls.

Initially, we proposed percutaneous drainage of the biloma under ultrasound (USG) guidance (Figure 3) to control the collection and reduce the risk of infection prior to definitive biliary decompression, which was performed without complications, yielding bile-stained fluid.

**Figure 3:**
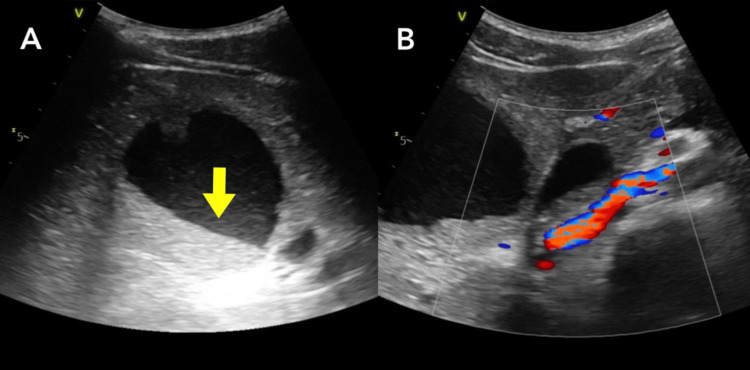
Abdominal ultrasound. A: Fluid content within the biloma (yellow arrow). B: Percutaneous drainage of the biloma under ultrasound guidance.

After drainage of the collection, the patient underwent ERCP to relieve the bile duct obstruction (Figure 4). The procedure confirmed significant bile duct stenosis approximately 4 cm above the papilla, which was impassable with a guidewire. A biliary sphincterotomy was performed, and a pancreatic stent was placed.

**Figure 4:**
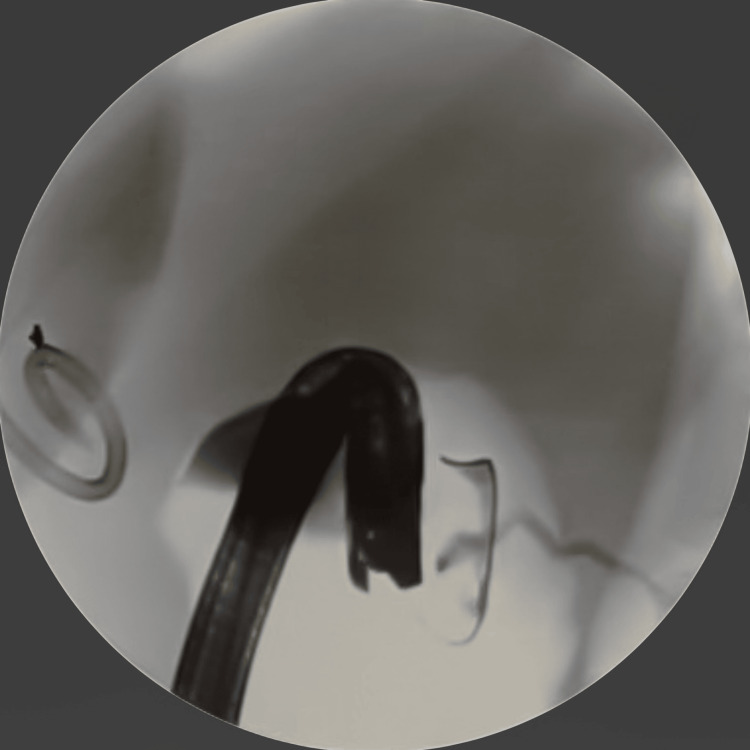
Endoscopic retrograde cholangiopancreatography (ERCP). ERCP was performed to relieve bile duct obstruction, confirming a significant stenosis approximately 4 cm above the papilla.

On the first postoperative day after ERCP, the patient presented with episodes of vomiting and malaise. He was pale (3+/4), jaundiced (1+/4), and dehydrated (3+/4), with a blood pressure of 70/50 mmHg. Laboratory findings (Table 1) revealed elevated pancreatic amylase levels (1399 U/L), decreased hemoglobin levels (6.8 g/dL), and acute kidney injury (AKI) classified as KDIGO (Kidney Disease: Improving Global Outcomes) stage III. The combination of the clinical presentation and elevated pancreatic amylase levels supported the diagnosis of severe acute pancreatitis, while the drop in hematocrit levels and low blood pressure suggested post-ERCP hemorrhage.

**Table 1:** Laboratory results obtained approximately 24 hours after ERCP indicated hemorrhagic shock, acute kidney injury, and acute pancreatitis. Laboratory findings obtained 24 hours after endoscopic retrograde cholangiopancreatography (ERCP), demonstrating severe anemia consistent with hemorrhagic shock (hemoglobin = 6.8 g/dL, hematocrit = 18.8%, RBC = 2.26 ×10⁶/mm³), stage 3 acute kidney injury (creatinine = 4.2 mg/dL, urea = 187.6 mg/dL), and acute pancreatitis (amylase = 1399 U/L, WBC at 29,850/mm³ with neutrophilia and bandemia). Additional abnormalities included metabolic acidosis with partial respiratory compensation (pH = 7.37, HCO₃⁻ = 18.4 mmol/L, BE = –5.6 mmol/L), hyperbilirubinemia with mixed pattern (total bilirubin = 16.1 mg/dL; direct = 8.57 mg/dL), and mild coagulopathy (INR = 1.43).

Laboratory findings		
Test	Result	Reference range
Arterial blood gas		
pH	7.376	7.35 - 7.45
Partial pressure of carbon dioxide (pCO2)	32.2 mmHg	35 - 45 mmHg
Partial pressure of oxygen (pO2)	122.3 mmHg	80 - 100 mmHg
Oxygen saturation (sO2)	98.4%	95 - 100%
Bicarbonate (HCO3)	18.4 mmol/L	22 - 28 mmol/L
Total carbon dioxide (TCO2)	19.4 mmol/L	23 - 30 mmol/L
Base excess (BE)	-5.6 mmol/L	-2 to +2 mmol/L
Lactate	0.5 mmol/L	0.5 - 2.2 mmol/L
Renal function		
Creatinine	4.2 mg/dL	0.6 - 1.3 mg/dL
Urea	187.6 mg/dL	10 - 50 mg/dL
Complete blood count (CBC)		
Red blood cells (RBC)	2.26 million/mm³	4.52 - 5.9 (M)/4.1 - 5.1 (F)
Hemoglobin	6.8 g/dL	13.3 - 17.5 (M)/12.3 - 15.3 (F)
Hematocrit	18.8%	41.5 - 51.4 (M)/35.9 - 45.6 (F)
White blood cells (WBC)	29,850/mm³	4,500 - 11,000/mm³
Neutrophils	26,626/mm³ (89.2%)	1,900 - 7,700/mm³ (40 - 75%)
Band neutrophils	2,388/mm³ (8%)	0 - 700/mm³ (1 - 3%)
Eosinophils	0/mm³ (0%)	40 - 450/mm³ (0 - 4%)
Basophils	179/mm³ (0.6%)	0 - 200/mm³ (0 - 1%)
Lymphocytes	1,104/mm³ (3.7%)	1,000 - 4,000/mm³ (20 - 45%)
Monocytes	1,940/mm³ (6.5%)	90 - 800/mm³ (2 - 10%)
Platelets	185,000/mm³	150,000 - 450,000 /mm³
Coagulation		
International normalized ratio (INR)	1.43	0.95 - 1.20
Liver function		
Total bilirubin	16.10 mg/dL	0.2 - 1.2 mg/dL
Direct bilirubin	8.57 mg/dL	0.0 - 0.3 mg/dL
Indirect bilirubin	7.53 mg/dL	<1.0 mg/dL
Aspartate aminotransferase (AST)	70 U/L	0 - 40 U/L
Alanine aminotransferase (ALT)	55 U/L	0 - 40 U/L
Gamma-glutamyl transferase (GGT)	42 U/L	9 - 36 U/L
Alkaline phosphatase (ALP)	142 U/L	30 - 120 U/L
Amylase	1399 U/L	23 - 85 U/L

Due to clinical deterioration and a sustained drop in hemoglobin levels, the patient required ICU support and endoscopic management. Esophagogastroduodenoscopy (EGD) revealed bleeding from the duodenal papilla, with a lesion at the apex of the papillotomy. Endoscopic hemostasis of the duodenal papilla was successfully performed.

Due to persistent jaundice, a second ERCP was performed the following day. During this procedure, a biliary prosthesis was successfully inserted, allowing effective bile duct drainage. Hemostasis of the duodenal papilla was achieved using four clips at the edge of the papillotomy (Figure 5). Following the procedure, a significant reduction in bilirubin levels was observed, and the patient’s clinical condition improved. An X-ray confirmed the correct positioning of the biliary prosthesis (Figure 6). The patient was subsequently transferred to the ward.

**Figure 5:**
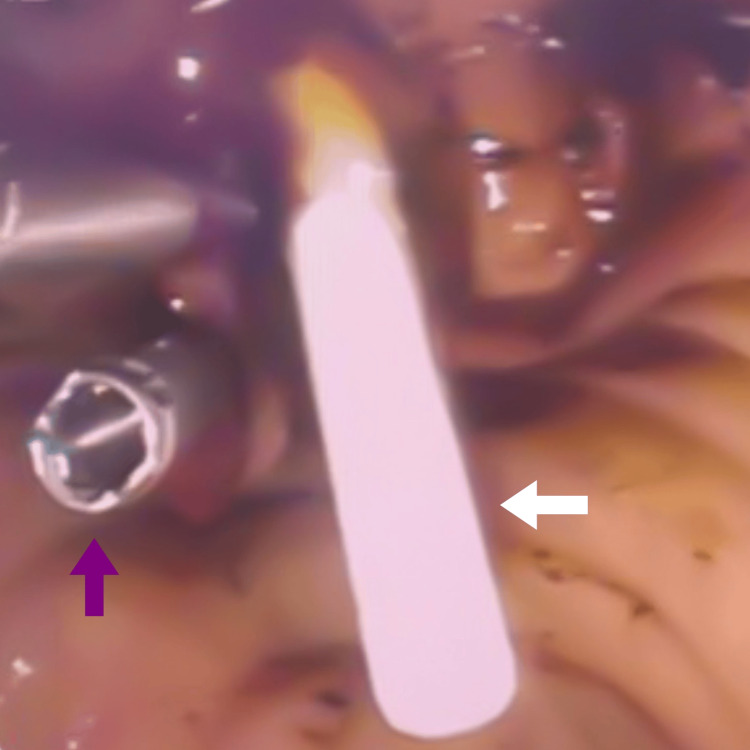
Esophagogastroduodenoscopy (EGD). Biliary prosthesis (white arrow), with hemostasis of the duodenal papilla achieved using clips (purple arrow) placed at the edge of the papillotomy.

**Figure 6:**
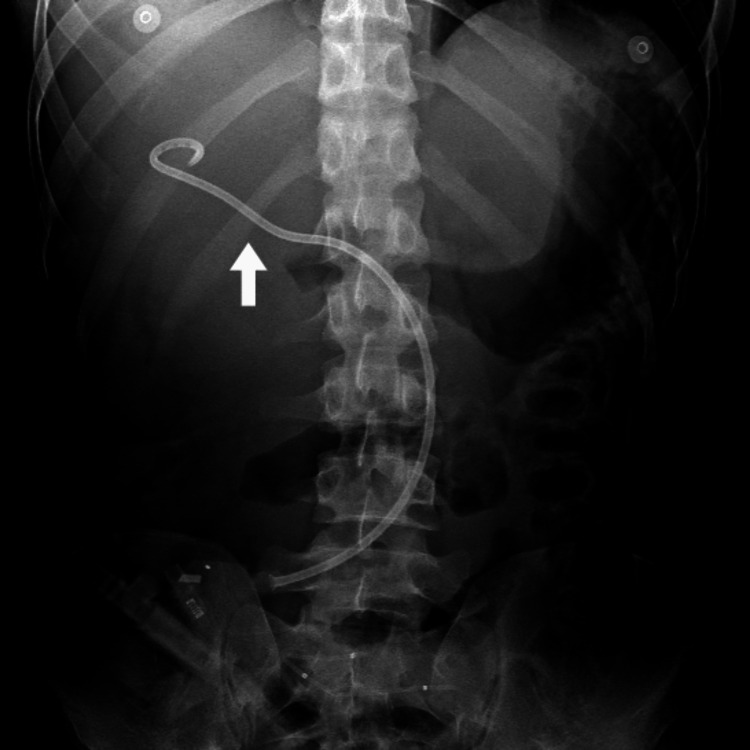
Abdominal X-ray. Biliary prosthesis (white arrow) facilitating bile duct drainage.

After transfer to the ward, the patient maintained a reduction in liver enzyme levels and remained hemodynamically stable. However, he remained hospitalized for ongoing monitoring and supportive care. During the same hospitalization, 17 days after prosthesis placement, the patient developed leukocytosis and fever. The infectious workup included a chest X-ray, which revealed a significant pleural effusion associated with consolidation at the right lung base (Figure 7).

**Figure 7:**
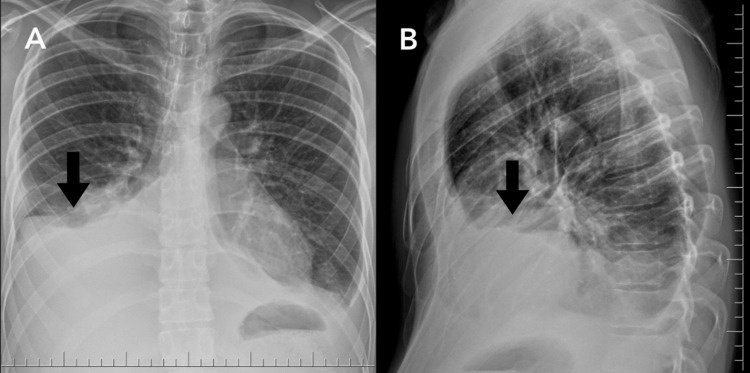
Chest X-ray. Posteroanterior (A) and lateral (B) radiographs showing a large right-sided pleural effusion (black arrow).

The patient underwent thoracostomy with closed pleural drainage due to a large symptomatic pleural effusion associated with fever, leukocytosis, and respiratory symptoms, along with radiological evidence of consolidation at the right lung base. Differential diagnoses, including bilothorax and sympathetic effusion, were considered. However, the clinical and imaging findings were more consistent with a parapneumonic effusion.

The patient received antibiotic therapy with piperacillin-tazobactam, resulting in clinical and radiological improvement, allowing for subsequent chest tube removal. Since then, he has been under outpatient follow-up, undergoing serial dilation of the common bile duct with progressively larger prostheses, with a satisfactory clinical response. No new hepatic collections or other clinical complications have been observed.

## Discussion

In recent decades, the introduction of damage control strategies and nonoperative management for hemodynamically stable patients with blunt hepatic trauma has significantly reduced mortality rates. However, this approach has been associated with an increased incidence of hepatic complications, as reported in the literature, including biliary tree rupture, bile leakage, biloma formation, and bile duct strictures, as demonstrated in our case [1,2].

Bile duct strictures represent a significant clinical challenge in hepatobiliary practice. This condition is characterized by narrowing of the bile ducts, which can result in partial or complete obstruction of bile flow [3].

While bile duct strictures are most commonly secondary to biliary tract surgeries, other causes include fibrosis due to chronic pancreatitis, gastrointestinal tract neoplasms, primary sclerosing cholangitis, or abdominal trauma. In the present case, blunt abdominal trauma was the underlying cause of biliary stenosis. Such injuries lead to inflammatory changes that can alter the integrity of the bile duct wall, particularly at the hepatic hilum. If not identified or adequately treated, they may result in serious complications, such as cholangitis, biliary cirrhosis, portal hypertension, and even death [3,4].

Traumatic injuries to the biliary tract can involve various structures. The most commonly affected are the extrahepatic bile ducts, due to their anatomical fixation sites; the intrahepatic bile ducts, often associated with parenchymal lacerations; and the gallbladder, especially when distended [1].

The pathogenesis of traumatic bile duct injury and stricture formation is multifactorial, typically involving bile duct rupture, inflammation, ductal compression by hematomas, or ischemic injury caused by interruption of the blood supply to the duct. In cases of direct traumatic bile duct injury, partial or total destruction of the duct may occur, culminating in a scarring process and subsequent stenosis. Additionally, the inflammatory response to trauma, including hematoma and abscess formation, can result in fibrosis around the bile ducts, leading to narrowing. Finally, vascular injuries may compromise blood supply to the bile ducts, causing necrosis and subsequent stenosis [5].

The presence of jaundice after blunt trauma should raise suspicion for possible injuries to the biliary tree. Clinical manifestations are often nonspecific, including progressive abdominal discomfort, nausea, vomiting, fever, and persistent ileus, but these may serve as important indicators of biliary involvement. Morbidity in such cases can reach up to 40%, largely due to the delayed onset of clinical signs and the frequent development of complications such as hemorrhage, infection, and abdominal compartment syndrome. Even when timely intervention is undertaken, postoperative complications may arise, including anastomotic leakage, recurrent cholangitis with or without stricture formation, biliary cirrhosis, and portal hypertension [2]. Risk factors for developing biliary tract injuries include hepatic injury, central parenchymal injury, and elevated serum levels of bilirubin and gamma-glutamyl transferase (GGT) [1].

Diagnosing these injuries is challenging and requires a high degree of clinical suspicion, as conventional imaging tests are generally nonspecific. Non-invasive diagnostic tools, such as ultrasound, CT, and MRCP, are commonly used to assess the level of biliary obstruction [4,5].

The treatment of traumatic biliary injuries is significantly more challenging than that of iatrogenic biliary injuries due to the presence of associated injuries to adjacent organs and delayed diagnosis. Effective management requires close interdisciplinary collaboration between surgery, interventional gastroenterology, and interventional radiology [6]. Endoscopic therapy, performed via ERCP, is considered the first-line treatment because of its high success rate and low risk of significant complications. This technique facilitates imaging documentation, histopathological diagnosis, and definitive intervention, with outcomes comparable to or even better than surgical treatment [1,6].

During the procedure, the guidewire should be carefully advanced into the intrahepatic biliary tree to allow stricture dilation and subsequent placement of either a plastic or metal prosthesis. Hydrophilic guidewires are generally preferred initially, as they are more effective in traversing tight or complex strictures. Dilation should be performed progressively using dilating catheters or radial balloons, which facilitate the placement of stents. The success of the repair may be influenced by several factors, including the timing of intervention, the presence of infection, associated vascular injury, the extent of the stricture, and the surgical technique employed [4,7].

Adverse effects of ERCP include acute pancreatitis, hemorrhage, perforation, cholangitis, and stenosis or dysfunction of the sphincter of Oddi. Acute pancreatitis is the most common complication and the leading cause of ERCP-related mortality, with an incidence ranging from 1.6% to 15% (most studies report a mean of 3-5%) and a mortality rate of 0.7% [7]. It is diagnosed by a serum amylase level more than three times the reference value, accompanied by a compatible clinical presentation, often requiring prolonged hospitalization or readmission. In uncertain cases, imaging studies can aid in confirming the diagnosis [7,8].

Hemorrhage is another serious complication of ERCP, often associated with endoscopic sphincterotomy, which is essential for performing therapeutic maneuvers during the procedure. Clinically significant bleeding, as seen in our case, occurs in approximately 0.3% to 2% of cases [7]. Hemorrhage control can be achieved through adrenaline injection at the bleeding site, electrocoagulation, or hemostatic clips, which are particularly effective for localized vessels and focal bleeding [7,8].

Delayed biliary stenosis following blunt abdominal trauma is an uncommon but recognized complication, typically presenting weeks to months after the initial injury, as observed in the present case. Similar cases reported in the literature suggest that progressive inflammation, fibrosis, and ischemic injury contribute to late stricture formation. In this case, the occurrence of severe complications following ERCP, including acute pancreatitis and hemorrhagic shock, may be explained by difficult cannulation and manipulation in the setting of a tight and possibly impassable stricture, which are known risk factors for post-ERCP adverse events. In such scenarios, alternative therapeutic approaches, including endoscopic ultrasound (EUS)-guided biliary drainage, may be considered, particularly when conventional ERCP is technically challenging or unsuccessful. Prognosis in these patients depends on early recognition, effective biliary decompression, and adequate management of complications. Long-term outcomes are generally favorable with endoscopic treatment, although repeated interventions may be required, as demonstrated in this case.

## Conclusions

Bile duct strictures are rare complications of blunt abdominal trauma and may lead to significant morbidity if not promptly recognized. This case highlights the importance of maintaining a high index of suspicion for biliary tract injury in patients presenting with delayed symptoms after trauma. Minimally invasive management with ERCP and guided drainage can be effective. However, it is not without risk, as demonstrated by the severe complications observed in this case, including acute pancreatitis, hemorrhagic shock, and pleural effusion.

This case underscores the need for careful patient selection and procedural planning, particularly when biliary stenosis is severe or impassable, as endoscopic interventions may carry increased risk. Additionally, multidisciplinary management and close clinical monitoring are essential to optimize outcomes.
